# Efficient Implementation of Penalized Regression for Genetic Risk Prediction

**DOI:** 10.1534/genetics.119.302019

**Published:** 2019-02-26

**Authors:** Florian Privé, Hugues Aschard, Michael G. B. Blum

**Affiliations:** * Laboratoire TIMC-IMAG, UMR 5525, University of Grenoble Alpes, CNRS, 38700 La Tronche, France; † Centre de Bioinformatique, Biostatistique et Biologie Intégrative (C3BI), Institut Pasteur, 75015 Paris, France

**Keywords:** polygenic risk scores, SNP, LASSO, genomic prediction, GenPred, shared data resources

## Abstract

Polygenic Risk Scores (PRS) combine genotype information across many single-nucleotide polymorphisms (SNPs) to give a score reflecting the genetic risk of developing a disease. PRS might have a major impact on public health, possibly allowing for screening campaigns to identify high-genetic risk individuals for a given disease. The “Clumping+Thresholding” (C+T) approach is the most common method to derive PRS. C+T uses only univariate genome-wide association studies (GWAS) summary statistics, which makes it fast and easy to use. However, previous work showed that jointly estimating SNP effects for computing PRS has the potential to significantly improve the predictive performance of PRS as compared to C+T. In this paper, we present an efficient method for the joint estimation of SNP effects using individual-level data, allowing for practical application of penalized logistic regression (PLR) on modern datasets including hundreds of thousands of individuals. Moreover, our implementation of PLR directly includes automatic choices for hyper-parameters. We also provide an implementation of penalized linear regression for quantitative traits. We compare the performance of PLR, C+T and a derivation of random forests using both real and simulated data. Overall, we find that PLR achieves equal or higher predictive performance than C+T in most scenarios considered, while being scalable to biobank data. In particular, we find that improvement in predictive performance is more pronounced when there are few effects located in nearby genomic regions with correlated SNPs; for instance, in simulations, AUC values increase from 83% with the best prediction of C+T to 92.5% with PLR. We confirm these results in a data analysis of a case-control study for celiac disease where PLR and the standard C+T method achieve AUC values of 89% and of 82.5%. Applying penalized linear regression to 350,000 individuals of the UK Biobank, we predict height with a larger correlation than with the best prediction of C+T (∼65% instead of ∼55%), further demonstrating its scalability and strong predictive power, even for highly polygenic traits. Moreover, using 150,000 individuals of the UK Biobank, we are able to predict breast cancer better than C+T, fitting PLR in a few minutes only. In conclusion, this paper demonstrates the feasibility and relevance of using penalized regression for PRS computation when large individual-level datasets are available, thanks to the efficient implementation available in our R package bigstatsr.

## Abstract (precis)

Polygenic risk scores (PRS) combine many single-nucleotide polymorphisms into a score reflecting the genetic risk of developing a disease. Privé, Aschard, and Blum present an efficient implementation of penalized logistic regression...

POLYGENIC risk scores (PRS) combine genotype information across many single-nucleotide polymorphisms (SNPs) to give a score reflecting the genetic risk of developing a disease. PRS are useful for genetic epidemiology when testing polygenicity of diseases and finding a common genetic contribution between two diseases (Purcell *et al.* 2009). Personalized medicine is another major application of PRS. Personalized medicine envisions to use PRS in screening campaigns in order to identify high-risk individuals for a given disease (Chatterjee *et al.* 2016). As an example of practical application, targeting screening of men at higher polygenic risk could reduce the problem of overdiagnosis and lead to a better benefit-to-harm balance in screening for prostate cancer (Pashayan *et al.* 2015). However, in order to be used in clinical settings, PRS should discriminate well enough between cases and controls. For screening high-risk individuals and for presymptomatic diagnosis of the general population, it is suggested that, for a 10% disease prevalence, the AUC must be >75% and 99%, respectively (Janssens *et al.* 2007).

Several methods have been developed to predict disease status, or any phenotype, based on SNP information. A commonly used method often called “P+T” or “C+T” (which stands for “Clumping and Thresholding”) is used to derive PRS from results of Genome-Wide Association Studies (GWAS) (Wray *et al.* 2007; Evans *et al.* 2009; Purcell *et al.* 2009; Chatterjee *et al.* 2013; Dudbridge 2013). This technique uses GWAS summary statistics, allowing for a fast implementation of C+T. However, C+T also has several limitations; for instance, previous studies have shown that predictive performance of C+T is very sensitive to the threshold of inclusion of SNPs, depending on the disease architecture (Ware *et al.* 2017). In parallel, statistical learning methods have also been used to derive PRS for complex human diseases by jointly estimating SNP effects. Such methods include joint logistic regression, Support Vector Machine (SVM) and random forests (Wei *et al.* 2009; Abraham *et al.* 2012, 2014; Botta *et al.* 2014; Okser *et al.* 2014; Lello *et al.* 2018; Mavaddat *et al.* 2019). Finally, Linear Mixed-Models (LMMs) are another widely used method in fields such as plant and animal breeding, or for predicting highly polygenic quantitative human phenotypes such as height (Yang *et al.* 2010). Yet, predictions resulting from LMM, known *e.g.*, as “gBLUP,” have not proven as efficient as other methods for predicting several complex diseases based on genotypes [see table 2 of Abraham *et al.* (2013)].

We recently developed two R packages, bigstatsr and bigsnpr, for efficiently analyzing large-scale genome-wide data (Privé *et al.* 2018). Package bigstatsr now includes an efficient algorithm with a new implementation for computing sparse linear and logistic regressions on huge datasets as large as the UK Biobank (Bycroft *et al.* 2018). In this paper, we present a comprehensive comparative study of our implementation of penalized logistic regression (PLR), which we compare to the C+T method and the T-Trees algorithm, a derivation of random forests that has shown high predictive performance (Botta *et al.* 2014). In this comparison, we do not include any LMM method, yet, L2-PLR should be very similar to LMM methods. Moreover, we do not include any SVM method because it is expected to give similar results to logistic regression (Abraham *et al.* 2012). For C+T, we report results for a large grid of hyper-parameters. For PLR, the choice of hyper-parameters is included in the algorithm so that we report only one model for each simulation. We also use a modified version of PLR in order to capture not only linear effects, but also recessive and dominant effects.

To perform simulations, we use real genotype data and simulate new phenotypes. In order to make our comparison as comprehensive as possible, we compare different disease architectures by varying the number, size and location of causal effects as well as disease heritability. We also compare two different models for simulating phenotypes, one with additive effects only, and one that combines additive, dominant and interaction-type effects. Overall, we find that PLR achieves higher predictive performance than C+T except in highly underpowered cases (AUC values lower than 0.6), while being scalable to biobank data.

## Materials and Methods

### Genotype data

We use real genotypes of European individuals from a case-control study for celiac disease (Dubois *et al.* 2010). This dataset is presented in Supplemental Material, Table S1. Details of quality control and imputation for this dataset are available in Privé *et al.* (2018). For simulations presented later, we first restrict this dataset to controls from UK in order to remove the genetic structure induced by the celiac disease status and population structure. This filtering process results in a sample of 7100 individuals (see supplemental notebook “preprocessing”). We also use this dataset for real data application, in this case keeping all 15,155 individuals (4496 cases and 10,659 controls). Both datasets contain 281,122 SNPs.

### Simulations of phenotypes

We simulate binary phenotypes using a Liability Threshold Model (LTM) with a prevalence of 30% (Falconer 1965). We vary simulation parameters in order to match a range of genetic architectures from low to high polygenicity. This is achieved by varying the number of causal variants and their location (30, 300, or 3000 anywhere in all 22 autosomal chromosomes or 30 in the HLA region of chromosome 6), and the disease heritability $h^{2}$ (50 or 80%). Liability scores are computed either from a model with additive effects only (“ADD”) or a more complex model that combines additive, dominant and interaction-type effects (“COMP”). For model “ADD,” we compute the liability score of the *i*-th individual as

$$y_{i}=\sum_{j\in S_{\text{causal}}}w_{j}\cdot \tilde{G_{i,j}}+\epsilon_{i},$$

where $S_{\text{causal}}$ is the set of causal SNPs, $w_{j}$ are weights generated from a Gaussian distribution $N\left(0,h^{2}/\left|S_{\text{causal}}\right|\right)$ or a Laplace distribution $Laplace\left(0,\sqrt{h^{2}/\left(2\left|S_{\text{causal}}\right|\right)}\right)$, $G_{i,j}$ is the allele count of individual *i* for SNP *j*, $\tilde{G_{i,j}}$ corresponds to its standardized version (zero mean and unit variance for all SNPs), and ϵ follows a Gaussian distribution $N\left(0,1-h^{2}\right)$. For model “COMP,” we simulate liability scores using additive, dominant and interaction-type effects (see Supplemental Materials).

We implement three different simulation scenarios, summarized in Table 1. Scenario N°1 uses the whole dataset (all 22 autosomal chromosomes – 281,122 SNPs) and a training set of size 6000. For each combination of the remaining parameters, results are based on 100 simulations except when comparing PLR with T-Trees, which relies on five simulations only because of a much higher computational burden of T-Trees as compared to other methods. Scenario N°2 consists of 100 simulations per combination of parameters on a dataset composed of chromosome six only (18,941 SNPs). Reducing the number of SNPs increases the polygenicity (the proportion of causal SNPs) of the simulated models. Reducing the number of SNPs (*p*) is also equivalent to increasing the sample size (*n*) as predictive power increases as a function of n/p (Dudbridge 2013; Vilhjálmsson *et al.* 2015). For this scenario, we use the additive model only, but continue to vary all other simulation parameters. Finally, scenario N°3 uses the whole dataset as in scenario N°1 while varying the size of the training set in order to assess how the sample size affects predictive performance of methods. A total of 100 simulations per combination of parameters are run using 300 causal SNPs randomly chosen on the genome.

**Table 1:** Summary of all simulations

Number of scenario	Dataset (number of SNPs)	Sample size of training set	Causal SNPs (number and location)	Distribution of effects	Heritability	Simulation model	Methods
1	All 22 chromosomes	6000	30 in HLA	Gaussian	0.5	ADD	C+T
			30 in all				PLR
	(281,122 SNPs)		300 in all	Laplace	0.8	COMP	PLR3
			3000 in all				(T-Trees)

2	Chromosome 6 only	—*^a^*	— *^a^*	— *^a^*	— *^a^*	ADD	C+T
	(18,941 SNPs)						PLR

3	All 22 chromosomes	1000	300 in all	— *^a^*	— *^a^*	— *^a^*	— *^a^*
	(281,122 SNPs)	2000					
		3000					
		4000					
		5000					

a Parameters are the same as the ones in the upper box.

### Predictive performance measures

In this study, we use two different measures of predictive accuracy. First, we use the Area Under the Receiver Operating Characteristic (ROC) Curve (AUC) (Lusted 1971; Fawcett 2006). In the case of our study, the AUC is the probability that the PRS of a case is greater than the PRS of a control. This measure indicates the extent to which we can distinguish between cases and controls using PRS. As a second measure, we also report the partial AUC for specificities between 90 and 100% (McClish 1989; Dodd and Pepe 2003). This measure is similar to the AUC, but focuses on high specificities, which is the most useful part of the ROC curve in clinical settings. When reporting AUC results of simulations, we also report maximum achievable AUC values of 84% and 94% for heritabilities of 50% and 80%, respectively. These estimates are based on three different yet consistent estimations (see Supplemental Materials).

### Methods compared

In this paper, we compare three different types of methods: the C+T method, T-Trees and PLR.

The C+T method directly derives PRS from the results of Genome-Wide Associations Studies (GWAS). In GWAS, a coefficient of regression (*i.e.*, the estimated effect size ${\hat{\beta }}_{j}$) is learned independently for each SNP *j* along with a corresponding *P*-value $p_{j}$. The SNPs are first clumped (C) so that there remain only loci that are weakly correlated with one another (this set of SNPs is denoted $S_{\text{clumping}}$). Then, thresholding (T) consists in removing SNPs with *P*-values larger than a user-defined threshold $p_{T}$. Finally, the PRS for individual *i* is defined as the sum of allele counts of the remaining SNPs weighted by the corresponding effect coefficients

$${\text{PRS}}_{\text{i}}=\sum_{\begin{array}{c} j\in S_{\text{clumping}}\\ p_{j}<p_{T} \end{array}}{\hat{\beta }}_{\text{j}}\cdot {\text{G}}_{\text{i},\text{j}},$$

where ${\hat{\beta }}_{j}$ $\left(p_{j}\right)$ are the effect sizes (*P*-values) learned from the GWAS. In this study, we mostly report scores for a clumping threshold at $r^{2}>0.2$ within regions of 500 kb, but we also investigate thresholds of 0.05 and 0.8. We report three different scores of prediction: one including all the SNPs remaining after clumping (denoted “C+T-all”), one including only the SNPs remaining after clumping and that have a *P*-value under the GWAS threshold of significance ($P<5\cdot 10^{-8}$, “C+T-stringent”), and one that maximizes the AUC (“C+T-max”) for 102 *P*-value thresholds between 1 and $10^{-100}$ (Table S2). As we report the optimal threshold based on the test set, the AUC for “C+T-max” is an upper bound of the AUC for the C+T method. Here, the GWAS part uses the training set while clumping uses the test set (all individuals not included in the training set).

T-Trees (*Trees inside Trees*) is an algorithm derived from random forests (Breiman 2001) that takes into account the correlation structure among the genetic markers implied by linkage disequilibrium (Botta *et al.* 2014). We use the same parameters as reported in table 4 of Botta *et al.* (2014), except that we use 100 trees instead of 1000. Using 1000 trees provides a minimal increase of AUC while requiring a disproportionately long processing time (*e.g.*, AUC of 81.5% instead of 81%, data not shown).

Finally, for PLR, we find regression coefficients $\beta_{0}$ and *β* that minimize the following regularized loss function

$$\begin{array}{l} L\left(\lambda ,\alpha \right)=-\underset{\text{Loss function}}{\underset{︸}{\sum_{i=1}^{n}\left(y_{i}\text{log}\left(z_{i}\right)+\left(1-y_{i}\right)\text{log}\left(1-z_{i}\right)\right)}}\\ \;\;\;\;\;\;\;\;\;\;\;\;\;\;\;\;\;\;\;\;\;\;\;+\underset{\text{Penalization}}{\underset{︸}{\lambda \left(\left(1-\alpha \right)\frac{1}{2}{\left|\left|\beta \right|\right|}_{2}^{2}+\alpha {\left|\left|\beta \right|\right|}_{1}\right)}}, \end{array}$$ (1)

where $z_{i}=1/\left(1+\text{exp}\left(-\left(\beta_{0}+x_{i}^{T}\beta \right)\right)\right)$, *x* denotes the genotypes and covariables (*e.g.*, principal components), *y* is the disease status to predict, *λ* and *α* are two regularization hyper-parameters that need to be chosen. Different regularizations can be used to prevent overfitting, among other benefits: the L2-regularization (“ridge,” Hoerl and Kennard (1970)) shrinks coefficients and is ideal if there are many predictors drawn from a Gaussian distribution (corresponds to α=0 in the previous equation); the L1-regularization (“lasso,” Tibshirani 1996) forces some of the coefficients to be equal to zero and can be used as a means of variable selection, leading to sparse models (corresponds to α=1); the L1- and L2-regularization (“elastic-net,” Zou and Hastie 2005) is a compromise between the two previous penalties and is particularly useful in the p≫n situation (*p* is the number of SNPs), or any situation involving many correlated predictors (corresponds to 0<α<1) (Friedman *et al.* 2010). In this study, we use a grid search over α∈{1,0.5,0.05,0.001}. This grid-search is directly embedded in our PLR implementation for simplicity. Using α=0.001 should result in a model very similar to gBLUP.

To fit PLR, we use an efficient algorithm (Friedman *et al.* 2010; Tibshirani *et al.* 2012; Zeng and Breheny 2017) from which we derived our own implementation in R package bigstatsr. This algorithm builds predictions for many values of λ, which is called a “regularization path.” To obtain an algorithm that does not require to choose this hyper-parameter λ, we developed a procedure that we call Cross-Model Selection and Averaging (CMSA, Figure S1). Because of L1-regularization, the resulting vector of estimated effect sizes is sparse. We refer to this method as “PLR” in the results section.

To capture recessive and dominant effects on top of additive effects in PLR, we use simple feature engineering: we construct a separate dataset with three times as many variables as the initial one. For each SNP variable, we add two more variables coding for recessive and dominant effects: one variable is coded 1 if homozygous variant and 0 otherwise, and the other is coded 0 for homozygous referent and 1 otherwise. We then apply our PLR implementation to this dataset with three times as many variables as the initial one; we refer to this method as “PLR3” in the rest of the paper.

### Evaluating predictive performance for celiac data

We use Monte Carlo cross-validation to compute AUC, partial AUC, the number of predictors, and execution time for the original Celiac dataset with the observed case-control status: we randomly split 100 times the dataset in a training set of 12,000 individuals and a test set composed of the remaining 3155 individuals.

### Data availability

Supplemental Data include a PDF with two sections of methods, two tables and eight figures. Supplemental data also include six HTML R notebooks including all code and results used in this paper, for reproducibility purposes, and available at https://figshare.com/articles/code/7178750. Additional analyses of the UK Biobank are available as three R scripts at https://figshare.com/articles/code_UKB/7531559. Results of simulations are available at https://figshare.com/articles/results_zip/7126964. A tutorial on how to start with R packages bigstatsr and bigsnpr is available at https://privefl.github.io/bigsnpr/articles/demo.html. The two R packages are available on GitHub. Supplemental material available at https://doi.org/10.25386/genetics.7851470.

## Results

### Joint estimation improves predictive performance

**Figure 1:**
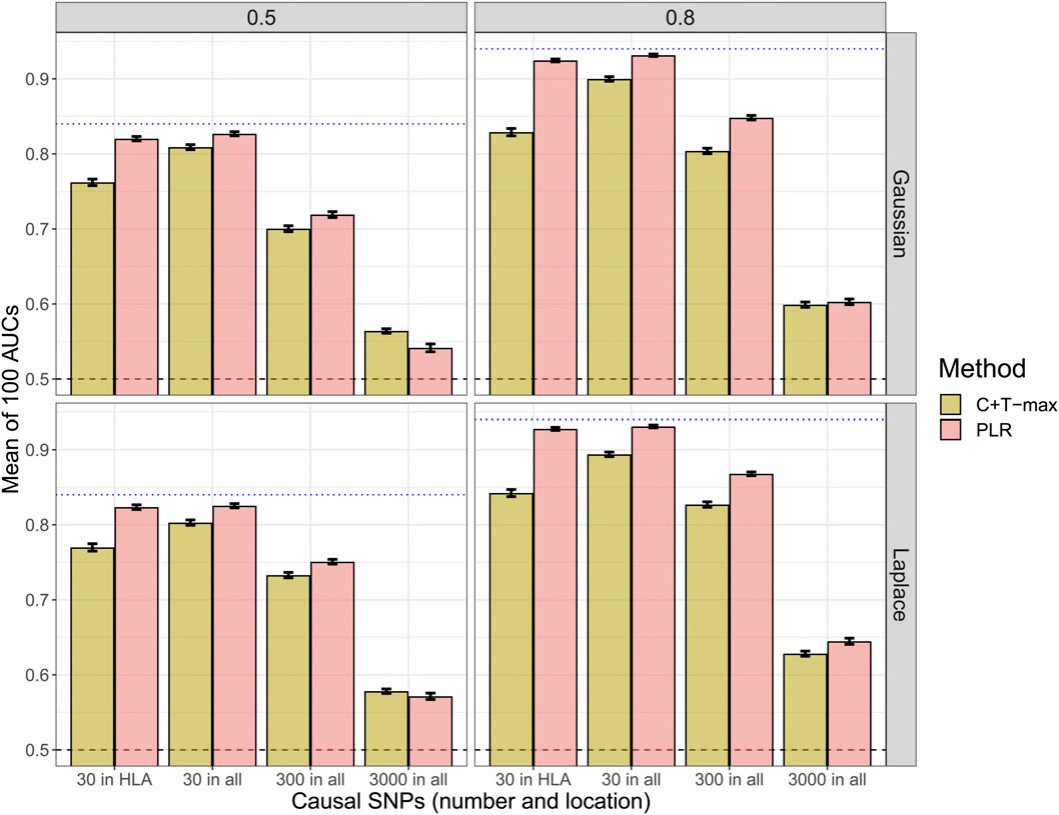
Main comparison of C+T and PLR when simulating phenotypes with additive effects (scenario N°1, model “ADD”). Mean AUC over 100 simulations for PLR and the maximum AUC reported with “C+T-max” (clumping threshold at $r^{2}>0.2$). Upper (lower) panels present results for effects following a Gaussian (Laplace) distribution, and left (right) panels present results for a heritability of 0.5 (0.8). Error bars are representing ±2SD of $10^{5}$ nonparametric bootstrap of the mean AUC. The blue dotted line represents the maximum achievable AUC.

We compared PLR with the C+T method using simulations of scenario N°1 (Table 1). When simulating a model with 30 causal SNPs and a heritability of 80%, PLR provides AUC of 93%, nearly reaching the maximum achievable AUC of 94% for this setting (Figure 1). Moreover, PLR consistently provides higher predictive performance than C+T across all scenarios considered, except in some cases of high polygenicity and small sample size, where all methods perform poorly (AUC values below 60% – Figure 1 and Figure 3). PLR provides particularly higher predictive performance than C+T when there are correlations between predictors, *i.e.*, when we choose causal SNPs to be in the HLA region. In this situation, the mean AUC reaches 92.5% for PLR and 84% for “C+T-max” (Figure 1). For the simulations, we do not report results in terms of partial AUC because partial AUC values have a Spearman correlation of 98% with the AUC results for all methods (Figure S3).

### Importance of hyper-parameters

In practice, a particular value of the threshold of inclusion of SNPs should be chosen for the C+T method, and this choice can dramatically impact the predictive performance of C+T. For example, in a model with 30 causal SNPs, AUC ranges from <60% when using all SNPs passing clumping to 90% *if* choosing the optimal *P*-value threshold (Figure S4).

**Figure 2:**
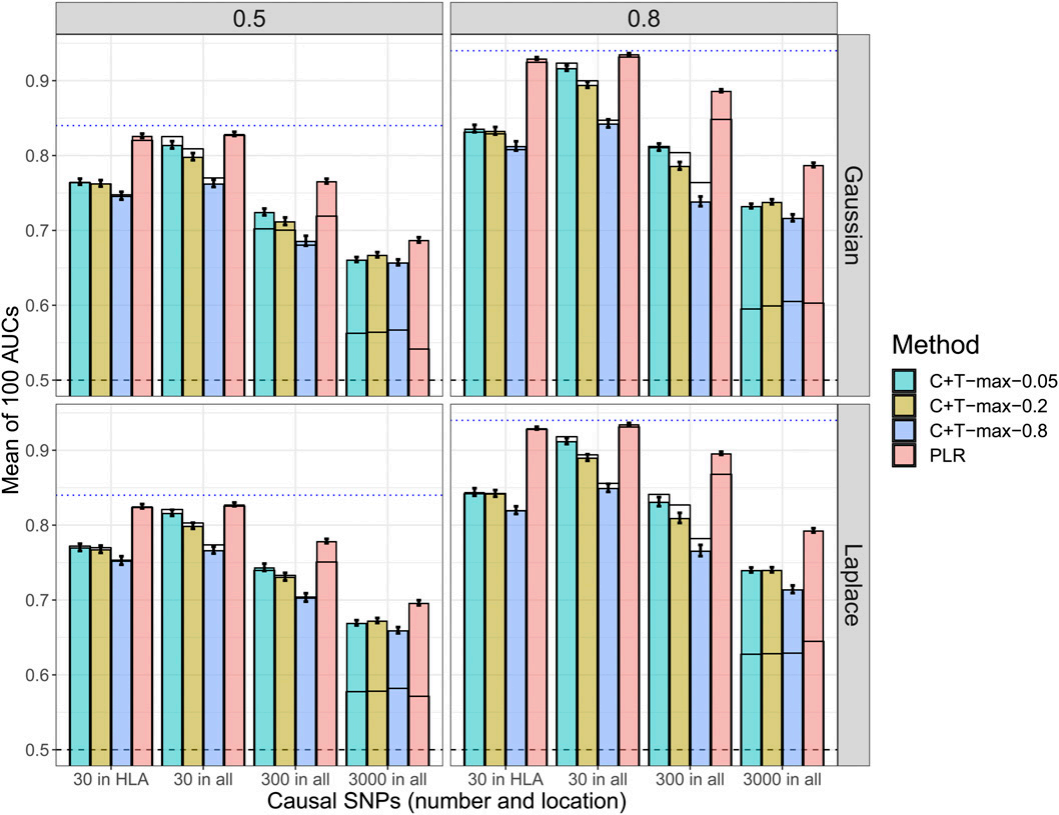
Comparison of methods when simulating phenotypes with additive effects and using chromosome six only (scenario N°2). Thinner lines represent results in scenario N°1. Mean AUC over 100 simulations for PLR and the maximum values of C+T for three different $r^{2}$ thresholds (0.05, 0.2, and 0.8) as a function of the number and location of causal SNPs. Upper (lower) panels present results for effects following a Gaussian (Laplace) distribution and left (right) panels present results for a heritability of 0.5 (0.8). Error bars representing ±2SD of $10^{5}$ nonparametric bootstrap of the mean AUC. The blue dotted line represents the maximum achievable AUC.

**Figure 3:**
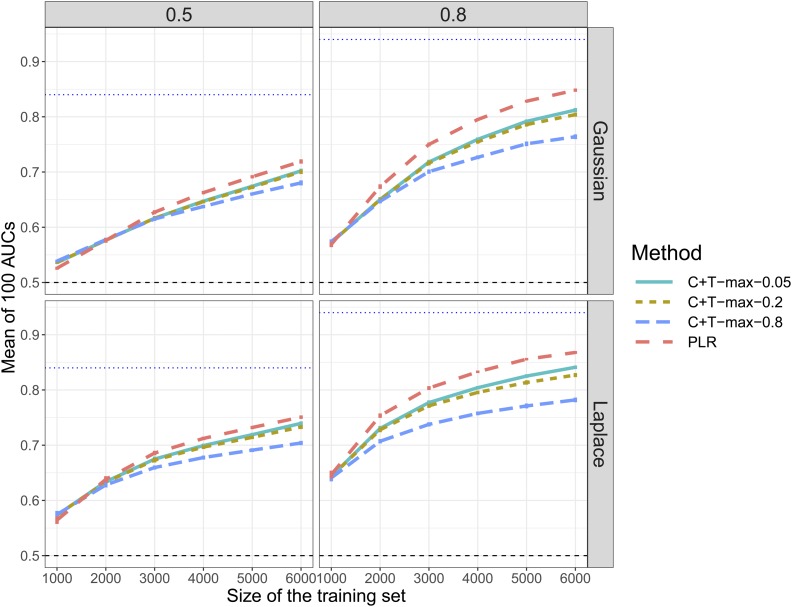
Comparison of methods when simulating 300 causal SNPs with additive effects and when varying sample size (scenario N°3). Mean AUC over 100 simulations for the maximum values of C+T for three different $r^{2}$ thresholds (0.05, 0.2, and 0.8) and PLR as a function of the training size. Upper (lower) panels are presenting results for effects following a Gaussian (Laplace) distribution and left (right) panels are presenting results for a heritability of 0.5 (0.8). Error bars represent ±2SD of $10^{5}$ nonparametric bootstrap of the mean AUC. The blue dotted line represents the maximum achievable AUC.

Concerning the $r^{2}$ threshold of the clumping step in C+T, we mostly used the common value of 0.2. Yet, using a more stringent value of 0.05 provides equal or higher predictive performance than using 0.2 in most of the cases we considered (Figure 2 and Figure 3).

Our implementation of PLR that automatically chooses hyper-parameter *λ* provides similar predictive performance than the best predictive performance of 100 models corresponding to different values of *λ* (Figure S8).

### Nonlinear effects

We tested the T-Trees method in scenario N°1. As compared to PLR, T-Trees perform worse in terms of predictive ability, while taking much longer to run (Figure S5). Even for simulations with model “COMP” in which there are dominant and interaction-type effects that T-Trees should be able to handle, AUC is still lower when using T-Trees than when using PLR (Figure S5).

We also compared the two PLRs in scenario N°1: PLR *vs.* PLR3 that uses additional features (variables) coding for recessive and dominant effects. Predictive performance of PLR3 are nearly as good as PLR when there are additive effects only (differences of AUC are always <2%) and can lead to significantly greater results when there are also dominant and interactions effects (Figures S6 and S7). For model “COMP,” PLR3 provides AUC values at least 3.5% higher than PLR, except when there are 3000 causal SNPs. Yet, PLR3 takes two to three times as much time to run and requires three times as much disk storage as PLR.

### Simulations varying number of SNPs and sample size

First, when reproducing simulations of scenario N°1 using chromosome six only (scenario N°2), the predictive performance of PLR always increase (Figure 2). There is a particularly large increase when simulating 3000 causal SNPs: AUC from PLR increases from 60% to nearly 80% for Gaussian effects and a disease heritability of 80%. On the contrary, when simulating only 30 or 300 causal SNPs with the corresponding dataset, AUC of “C+T-max” does not increase, and even decreases for a heritability of 80% (Figure 2). Second, when varying the training size (scenario N°3), we report an increase of AUC with a larger training size, with a faster increase of AUC for PLR as compared to “C+T-max” (Figure 3).

### Polygenic scores for celiac disease

Joint PLRs also provide higher AUC values for the Celiac data: 88.7% with PLR and 89.1% with PLR3 as compared to 82.5% with “C+T-max” (Figure S2 and Table 2). The relative increase in partial AUC, for specificities larger than 90%, is even larger (42 and 47%) with partial AUC values of 0.0411, 0.0426, and 0.0289 obtained with PLR, PLR3, and “C+T-max,” respectively. Moreover, logistic regressions use less predictors, respectively, at 1570, 2260, and 8360. In terms of computation time, we show that PLR, while learning jointly on all SNPs at once and testing four different values for hyper-parameter α, is almost as fast as the C+T method (190 *vs.* 130 sec), and PLR3 takes less than twice as long as PLR (296 *vs.* 190 sec).

**Table 2:** Results for the real celiac dataset

Method	AUC	pAUC	# predictors	Execution time (s)
C+T-max	0.825 (0.000664)	0.0289 (0.000187)	8360 (744)	130 (0.143)
PLR	0.887 (0.00061)	0.0411 (0.000224)	1570 (46.4)	190 (1.21)
PLR3	0.891 (0.000628)	0.0426 (0.000219)	2260 (56.1)	296 (2.03)

The results are averaged over 100 runs where the training step is randomly composed of 12,000 individuals. In the parentheses is reported the SD of $10^{5}$ bootstrap samples of the mean of the corresponding variable. Results are reported with three significant digits.

### Polygenic scores for the UK Biobank

We tested our implementation on 656K genotyped SNPs of the UK Biobank, keeping only Caucasian individuals and removing related individuals (excluding the second individual in each pair with a kinship coefficient >0.08). Results are presented in Table 3.

**Table 3:** Results for the UK Biobank

Trait	Method	*r* (women/men)	# Predictors	Execution time
Height	PLR	0.656/0.657	115,997	21 hr
Height	C+T-max	0.549/0.561	45,570	69 min

Trait	Method	AUC	# Predictors	Execution time
Breast cancer	PLR	0.598	2653	13 min
Breast cancer	C+T-max	0.589	21	15 hr

The sizes of training/test sets for height (resp. breast cancer) are 350,000/24,131 (resp. 150,000/38,628). For height, *r* (correlation between predicted and true heights) is reported within women/men separately; for breast cancer, AUC is reported.

Our implementation of L1-penalized linear regression runs in <1 day for 350K individuals (training set), achieving a correlation of >65.5% with true height for each sex in the remaining 24K individuals (test set). By comparison, the best C+T model achieves a correlation of 55% for women and 56% for men (in the test set), and the GWAS part takes 1 hr (for the training set). If using only the top 100,000 SNPs from a GWAS on the training set to fit our L1-PLR, correlation between predicted and true heights drops at 63.4% for women and 64.3% for men. Our L1-PLR on breast cancer runs in 13 min for 150K women, achieving an AUC of 0.598 in the remaining 39K women, while the best C+T model achieves an AUC of 0.589, and the GWAS part takes 15 hr.

## Discussion

### Joint estimation improves predictive performance

In this comparative study, we present a computationally efficient implementation of PLR. This model can be used to build PRS based on very large individual-level SNP datasets such as the UK biobank (Bycroft *et al.* 2018). In agreement with previous work (Abraham *et al.* 2013), we show that jointly estimating SNP effects has the potential to substantially improve predictive performance as compared to the standard C+T approach in which SNP effects are learned independently. PLR always outperforms the C+T method, except in some highly underpowered cases (AUC values always <0.6), and the benefits of using PLR are more pronounced with an increasing sample size or when causal SNPs are correlated with one another.

When there are many small effects and a small sample size, PLR performs worse than (the best result for) C+T. For example, this situation occurs when there are many causal variants (3K) to distinguish among many typed variants (280K) while using a small sample size (6K). In such underpowered scenarios, it is difficult to detect true causal variants, which makes PLR too conservative, whereas the best strategy is to include nearly all SNPs (Purcell *et al.* 2009).

When increasing sample size (scenario N°3), PLR achieves higher predictive performance than C+T and the benefits of using PLR over C+T increase with an increasing sample size (Figure 3). Moreover, when decreasing the search space (total number of candidate SNPs) in scenario N°2, we increase the proportion of causal variants and we virtually increase the sample size (Dudbridge 2013). In this scenario N°2, even when there are small effects and a high polygenicity (3000 causal variants out of 18,941), PLR gets a large increase in predictive performance, now consistently higher than C+T (Figure 2).

### Importance of hyper-parameters

The choice of hyper-parameter values is very important since it can greatly impact the performance of methods. In the C+T method, there are two main hyper-parameters: the $r^{2}$ and the $p_{T}$ thresholds that control how stringent are the C+T steps. For the clumping step, appropriately choosing the $r^{2}$ threshold is important. Indeed, on the one hand, choosing a low value for this threshold may discard informative SNPs that are correlated. On the other hand, when choosing a high value for this threshold, too much redundant information is included in the model, which adds noise to the PRS. Based on the simulations, we find that using a stringent threshold $\left(r^{2}=0.05\right)$ leads to higher predictive performance, even when causal SNPs are correlated. It means that, in most cases tested in this paper, avoiding redundant information in C+T is more important than including all causal SNPs. The choice of the $p_{T}$ threshold is also very important as it can greatly impact the predictive performance of the C+T method, which we confirm in this study (Ware *et al.* 2017). In this paper, we reported the maximum AUC of 102 different *P*-value thresholds, a threshold that should normally be learned on the training set only. To our knowledge, there is no clear standard on how to choose these two critical hyper-parameters for C+T. So, for C+T, we report the best AUC value on the test set, even if it leads to overoptimistic results for C+T as compared to PLR.

In contrast, for PLR, we developed an automatic procedure called CMSA that releases investigators from the burden of choosing hyper-parameter λ. Not only this procedure provides near-optimal results, but it also accelerates the model training thanks to the development of an early stopping criterion. Usually, cross-validation is used to choose hyper-parameter values and then the model is trained again with these particular hyper-parameter values (Hastie *et al.* 2008; Wei *et al.* 2013). Yet, performing cross-validation and retraining the model is computationally demanding; CMSA offers a less burdensome alternative. Concerning hyper-parameter α that accounts for the relative importance of the L1 and L2 regularizations, we use a grid search directly embedded in the CMSA procedure.

### Nonlinear effects

We also explored how to capture nonlinear effects. For this, we introduced a simple feature engineering technique that enables PLR to detect and learn not only additive effects, but also dominant and recessive effects. This technique improves the predictive performance of PLR when there are nonlinear effects in the simulations, while providing nearly the same predictive performance when there are additive effects only. Moreover, it also improves predictive performance for the celiac disease.

Yet, this approach is not able to detect interaction-type effects. In order to capture interaction-type effects, we tested T-Trees, a method that is able to exploit SNP correlations and interactions thanks to special decision trees (Botta *et al.* 2014). However, predictive performance of T-Trees are consistently lower than with PLR, even when simulating a model with dominant and interaction-type effects that T-Trees should be able to handle.

### Time and memory requirements

The computation time of our PLR implementation mainly depends on the sample size and the number of candidate variables (variables that are included in the gradient descent). Indeed, the algorithm is composed of two steps: first, for each variable, the algorithm computes an univariate statistic that is used to decide if the variable is included in the model (for each value of λ). This first step is very fast. Then, the algorithm iterates over a regularization path of decreasing values of λ, which progressively enables variables to enter the model (Figure S1). In the second step, the number of variables increases and computations stop when an early stopping criterion is reached (when prediction is getting worse on the corresponding validation set, see Figure S1).

For highly polygenic traits such as height and when using huge datasets such as the UK Biobank, the algorithm might iterate over >100,000 variables, which is computationally demanding. On the contrary, for traits like celiac disease or breast cancer that are less polygenic, the number of variables included in the model is much smaller so that fitting is very fast (only 13 min for 150K women of the UK Biobank for breast cancer).

Memory requirements are tightly linked to computation time. Indeed, variables are accessed in memory thanks to memory-mapping when they are used (Privé *et al.* 2018). When there is not enough memory left, the operating system (OS) frees some memory for new incoming variables. Yet, if too many variables are used in the gradient descent, the OS would regularly swap memory between disk and RAM, severely slowing down computations. A possible approach to reduce computational burden is to apply penalized regression on a subset of SNPs by prioritizing SNPs using univariate tests (GWAS computed from the same dataset). Yet, this strategy was shown to reduce predictive power (Abraham *et al.* 2013; Lello *et al.* 2018), which we also confirm in this paper. Indeed, when using only the 100K most significantly associated SNPs, correlation between predicted and true heights is reduced from 0.656/0.657 to 0.634/0.643 within women/men. A key advantage of our implementation of PLR is that prior filtering of variables is no more required for computational feasibility, thanks to the use of sequential strong rules and early stopping criteria.

### Limitations

Our approach has one major limitation: the main advantage of the C+T method is its direct applicability to summary statistics, allowing to leverage the largest GWAS results to date, even when individual cohort data cannot be merged because of practical or legal reasons. Our implementation of PLR does not allow yet for the analysis of summary data, but this represents an important future direction. The current version is of particular interest for the analysis of modern individual-level datasets including hundreds of thousands of individuals.

Finally, in this comparative study, we did not consider the problem of population structure (Vilhjálmsson *et al.* 2015; Márquez-Luna *et al.* 2017; Martin *et al.* 2017), and also did not consider nongenetic data such as environmental and clinical data (Van Vliet *et al.* 2012; Dey *et al.* 2013).

### Conclusions

In this comparative study, we have presented a computationally efficient implementation of PLR that can be used to predict disease status based on genotypes. A similar penalized linear regression for quantitative traits is also available in R package bigstatsr. Our approach solves the dramatic memory and computational burdens faced by standard implementations, thus allowing for the analysis of large-scale datasets such as the UK biobank (Bycroft *et al.* 2018).

We also demonstrated in simulations and real datasets that our implementation of penalized regressions is highly effective over a broad range of disease architectures. It can be appropriate for predicting autoimmune diseases with a few strong effects (*e.g.*, celiac disease), as well as highly polygenic traits (*e.g.*, standing height) provided that sample size is not too small. Finally, PLR as implemented in bigstatsr can also be used to predict phenotypes based on other omics data, since our implementation is not specific to genotype data.
